# Environmental Remediation to Address Childhood Lead Poisoning Epidemic due to Artisanal Gold Mining in Zamfara, Nigeria

**DOI:** 10.1289/ehp.1510145

**Published:** 2016-01-08

**Authors:** Simba Tirima, Casey Bartrem, Ian von Lindern, Margrit von Braun, Douglas Lind, Shehu Mohammed Anka, Aishat Abdullahi

**Affiliations:** 1 TerraGraphics International Foundation (TIFO), Moscow, Idaho, USA; 2 Environmental Science Program, University of Idaho, Moscow, Idaho, USA; 3 Zamfara Environmental Sanitation Agency (ZESA), Zamfara State, Nigeria

## Abstract

**Background:**

From 2010 through 2013, integrated health and environmental responses addressed an unprecedented epidemic lead poisoning in Zamfara State, northern Nigeria. Artisanal gold mining caused widespread contamination resulting in the deaths of > 400 children. Socioeconomic, logistic, and security challenges required remediation and medical protocols within the context of local resources, labor practices, and cultural traditions.

**Objectives:**

Our aim was to implement emergency environmental remediation to abate exposures to 17,000 lead poisoned villagers, to facilitate chelation treatment of children ≤ 5 years old, and to establish local technical capacity and lead health advocacy programs to prevent future disasters.

**Methods:**

U.S. hazardous waste removal protocols were modified to accommodate local agricultural practices. Remediation was conducted over 4 years in three phases, progressing from an emergency response by international personnel to comprehensive cleanup funded and accomplished by the Nigerian government.

**Results:**

More than 27,000 m3 of contaminated soils and mining waste were removed from 820 residences and ore processing areas in eight villages, largely by hand labor, and disposed in constructed landfills. Excavated areas were capped with clean soils (≤ 25 mg/kg lead), decreasing soil lead concentrations by 89%, and 2,349 children received chelation treatment. Pre-chelation geometric mean blood lead levels for children ≤ 5 years old decreased from 149 μg/dL to 15 μg/dL over the 4-year remedial program.

**Conclusions:**

The unprecedented outbreak and response demonstrate that, given sufficient political will and modest investment, the world’s most challenging environmental health crises can be addressed by adapting proven response protocols to the capabilities of host countries.

**Citation:**

Tirima S, Bartrem C, von Lindern I, von Braun M, Lind D, Anka SM, Abdullahi A. 2016. Environmental remediation to address childhood lead poisoning epidemic due to artisanal gold mining in Zamfara, Nigeria. Environ Health Perspect 124:1471–1478; http://dx.doi.org/10.1289/ehp.1510145

## Introduction

In March 2010, the humanitarian organization Médecins Sans Frontières (MSF) discovered an unprecedented epidemic of lead poisoning in remote villages of Zamfara State, Nigeria (MSF 2010b, 2010c). The first children brought to MSF clinics with convulsions and high fevers were treated for severe malaria and meningitis. As patients failed to recover, blood samples sent to a German laboratory (Labor Lademannbogen MVZ GmbH, Hamburg, Germany) confirmed lead poisoning (Greig et al. 2014). In May 2010, the U.S. Centers for Disease Control and Prevention (CDC) and the World Health Organization (WHO) dispatched medical and environmental investigators to collaborate with the Zamfara State and the Nigerian Federal Ministries of Health (ZMOH, FMOH) in assessing the epidemic (CDC 2010; WHO 2011). At CDC’s request, a U.S. firm, TerraGraphics Environmental Engineering (TG), accompanied the mission to investigate the potential for remediation (Brown MJ, Chief CDC Lead Poisoning and Prevention Program, personal communication, 11 May 2010). Extensive health and environmental assessments in two villages, Dareta and Yargalma, documented 163 deaths, including up to one-third of children < 5 years of age (Dooyema et al. 2012). Subsequent surveys of six additional villages showed that > 17,000 people were severely exposed and an estimated 400–500 children had died of acute lead poisoning (Dooyema et al. 2012; Greig et al. 2014; MSF 2010c; Thurtle et al. 2014; von Lindern et al. 2011; WHO 2011).

The source of lead contamination was prolific artisanal gold mining in response to high gold prices in 2009–2010. For several months, ore processing was conducted at sites within Dareta and Yargalma. Gold ore, sourced from mines throughout Zamfara State, is crushed by hand to gravel consistency using scrap hammers or mortars and pestles, ground to a fine powder in modified flour mills, sluiced by water to separate heavy particles, amalgamated with mercury, and burned over open flame to obtain a low-grade “sponge” gold that is sold to local traders for eventual refining for Dubai or Chinese markets. Grinding produces large quantities of dust, which settle on soils and surfaces. Sluicing results in water-source contamination and large piles of ore tailings. Because local religious and cultural practices include sequestration of married women, ore crushing, washing, and gold recovery were undertaken within the homes (“residential compounds”) to use the women’s labor. During the rapid increase in mining activities, a vein of ore exceeding 10% lead (Pb) was processed, resulting in severe residential exposures (Plumlee et al. 2013). By April 2010, local emirates suspected a link to children’s mortality and ordered artisanal ore-processing operations moved approximately 0.5 km from the villages. Extremely hazardous processing wastes and contaminated soils (> 3% Pb) remained in the compounds and public areas (von Lindern et al. 2011).

Blood lead levels, mortality and morbidity, and environmental lead concentrations observed in this epidemic are unprecedented (Moszynski 2010; Thurtle et al. 2014). In May 2010, the mortality rate in the initial villages surveyed was 25% of children ≤ 5 years; 59% of the surviving children ≤ 5 years were tested and 85% exceeded 65 μg/dL blood lead levels (BLLs) (Dooyema et al. 2012; Greig et al. 2014; Thurtle et al. 2014). The ensuing cleanup was one of the largest and most comprehensive undertaken by an African government (von Lindern et al. 2011; World Bank 2011). Several international organizations, Nigerian health authorities, and local civil and traditional governments collaborated to provide emergency medical, environmental, technical, and public health response. Due to the continuing mortality, the Zamfara State Ministry of Environment (ZMOE) and TG focused on emergency response, as MSF, ZMOH, and FMOH developed village clinics. Chelation therapy was limited to children ≤ 5 years of age and, from July through October 2010, was administered at inpatient clinics in 3–4 week cycles (Greig et al. 2014; Thurtle et al. 2014). All entities agreed that returning treated children to contaminated homes would compromise medical treatment. Coupled with local resistance to relocation, this required ZMOE and TG to remediate Dareta and Yargalma villages before inpatient discharge.

Cleanup of the villages presented numerous resource, logistic, cultural, institutional, and technical challenges. The remote area has little transportation or medical infrastructure. Villages are governed by overlapping civil, tribal, and Sharia law; exhibit sex-segregated social structure; suffer numerous endemic diseases with limited health care; and are supported by a workforce dependent on primitive tools and labor practices. Remediation activities were adapted to local technical and institutional capabilities from protocols developed at the Bunker Hill Superfund Site (BHSS), Idaho, USA (NRC 2005; Sheldrake and Stifelman 2003; U.S. EPA 2002; von Lindern et al. 2003, 2016). The mud-walled residential compounds could not be accessed by mechanical equipment and excavation was accomplished by modifying local hand labor agricultural practices. Remediation personnel adapted to sex, family, and village conventions. For example, initially, Sharia traditions limited compound access to men of the immediate family. Female remediation team members conducted initial characterization and interviews within the homes.

The overall lead health response program followed the model of the BHSS (i.e., curtailment of active sources, BLL testing and appropriate medical and environmental follow-up, remediation of residual soil contamination, and institutional controls to ensure sustainability of the remedy). Here we address adaption of the BHSS remediation and institutional controls to the Zamfara emergency. Details regarding the medical response can be found in studies by Greig et al. (2014) and Thurtle et al. (2014). The remediation program had two primary objectives: *a*) to reduce ongoing lead exposures and *b*) to develop in-country capacity to sustain the remedies and prevent future disasters. The first objective was 2-fold, both to facilitate the MSF/ZMOH chelation program for young children and to simultaneously decrease soil metals exposures for village residents. To sustain the remedies, three subobjectives included developing effective cleanup protocols that local communities could implement, building in-country technical capacity, and developing community awareness of the dangers of artisanal mining.

## Methods

The BHSS remediation strategy integrates contaminant removal and clean soil replacement, institutional controls, and lead health advocacy to reduce children’s lead intake to acceptable levels (NRC 2005; Sheldrake and Stifelman 2003; U.S. EPA 2002; von Lindern et al. 2003, 2011, 2016). These protocols were adapted for rural Zamfara using existing institutions, employing local labor, and using familiar labor practices, technology, and equipment. For example, traditional farming tools were used to excavate contaminated soils from compounds, which were not accessible by heavy equipment. ZMOE and local government areas (LGA) staff were trained to supervise labor, administer payrolls, and procure materials, supplies, and equipment. The international contingent provided quality assurance/quality control services and was responsible for database management to verify complete contaminant identification and removal.

The project was carried out in three phases: In Phase I, emergency response took place in Dareta and Yargalma villages in June–July 2010; these villages had an estimated combined population of 2,166 and are about 80 km apart, requiring separate operations bases. Phase II included remediation of Abare, Duza, Sunke, Tungar Daji, and Tungar Guru (October 2010–March 2011), with an estimated total population of 6,385 in a 1,400 km^2^ area requiring three operations bases. Phase III involved the remediation of Bagega (February–July 2013), with an estimated population of 7,323 and one base of operations (von Lindern et al. 2011). Remediation within each village was carried out in four steps: *a*) characterization, *b*) excavation of contaminated media, *c*) replacement with clean soils or concrete, and *d*) waste disposal (von Lindern et al. 2011). The social and technical context of the cleanup required adaptability, and remedial protocols were reevaluated and modified based on experience during all three phases. Examples include developing health messaging for both males and females as well as community engagement efforts; employing village tailors in the production, repair, and laundering of work uniforms; and having excavation hoes manufactured from scrap metal by local blacksmiths.

### Risk Assessment

Initial village surveys estimated that > 90% of ongoing lead exposure was attributable to ingestion of lead-contaminated soils and food (Dooyema et al. 2012; von Lindern et al. 2011). Primary sources were contaminated surface soils (generally < 5 cm depth) within the compounds, dusts on surfaces and soft materials (e.g., sleeping mats), and food preparation utensils used in ore processing. Several cubic meters of contaminated waste were identified in shady locations and near water sources throughout the villages where crushing, grinding, drying, sluicing, and storage of ores took place. Also, dangerous concentrations of arsenic, cadmium, mercury and manganese were observed, and these metals would be removed with the lead during remediation (Bartrem et al. 2014).

### Characterization

Common areas and residential compounds were assessed for lead and other metals *in situ* by modifying BHSS protocols for hand-held X-ray fluorescence (XRF) and by laboratory XRF of bulk and sieved samples of surface soils, dusts, and mining wastes (Bartrem et al. 2014; TerraGraphics 2009; von Lindern et al. 2011). Innov-X Alpha 4000 (Olympus), Innov-X (Olympus) Delta 4000, Innov-X (Olympus) DS 4000, and Niton XL3 (Thermo Fisher Scientific Inc.) models were used. A subset of soil samples was sent to four U.S. laboratories (U.S. EPA Region X, U.S. Geological Survey, University of Idaho Analytical Laboratory Services and Anatek Laboratories, Moscow, ID) for confirmatory analyses by inductively coupled mass spectrometry. Sharia traditions were amended by the local emirate, granting special permission to male staff and village workers to enter the compounds. Hand-drawn maps of homes and common areas included key features and XRF readings. Maps were modified to delineate areas requiring excavation, clean soil and concrete placement, and materials to be cleaned or replaced. Every residence and common area in all villages was sampled. Sample locations focused on the areas identified by the residents, with the objective of identifying locations requiring removal. Typically, 30–150 XRF readings were obtained within a single compound or common area. Sample-site selection was biased to avoiding false negatives (i.e., falsely identifying areas as not requiring removal).

### Excavation and Replacement

Before compound remediation, residents were required to remove, clean, and store utensils, bedding, and clothing. During Phase I, sleeping mats and carpets were collected, destroyed, and replaced. Areas with surface soil lead concentrations > 1,000 mg/kg were excavated to 5 cm depth. The exposed surface was retested by XRF and excavation continued until criteria (≤ 400 mg/kg Pb) were met (U.S. EPA 2008). Unexcavated areas with soil concentrations > 400 mg/kg and < 1,000 mg/kg were capped with ≥ 8 cm of clean soil obtained from landfill or borrow area excavations. Depending on drainage considerations and clean soil availability, unexcavated soils with ≤ 400 mg/kg Pb were covered with clean soil at the discretion of the project manager. All clean soils were confirmed ≤ 25 mg/kg lead by XRF. No delineation of the clean/contaminated soil interface was provided because foot and animal traffic quickly compacted the surface. Local agricultural hoes were used to scrape the surface backwards (to prevent recontamination) from the walls inward, starting at the rear of each compound and ending at the main entrance. If needed, interior compound walls were brushed to remove contaminated dust before excavation. Contaminated cement floors were capped with new concrete. Excavated soils were shoveled into grain sacks, removed by wheelbarrow, and trucked to landfills by village laborers. Heavy equipment was leased from foreign mining companies operating in the region to construct the waste disposal facilities and clean soil sources, and to excavate public areas. Landfill and health and safety protocols are described below.

In Phases I and II, most exterior removals were done by hand labor due to stability concerns for the mud walls surrounding the compounds and a lack of suitable mechanized equipment. In Phase III, the government obtained small skid-steer loaders that could safely maneuver the narrow streets, facilitating exterior area excavations and waste disposal. Both heavy equipment and hand labor were used to clean several contaminated human-made village ponds. By long-standing practice, village residents had constructed ponds to provide water for livestock and for clay to make bricks for residential compounds in the dry season. These ponds had been exploited for ore sluicing/amalgamation and were severely contaminated with lead and mercury. Bricks produced from the contaminated muck often had lead concentrations exceeding 1%. All bricks, mortar, and plaster were tested by XRF. Most contaminated bricks were identified before being used and were purchased by the ZMOE from the home owner and disposed of in landfills. A small number of contaminated bricks and clay plaster that had been incorporated in homes were capped or removed. The ponds were excavated when completely dry in late February 2011 and April 2013, and were closed or lined with clean soil. Clean soil was delivered to the villages for brickmaking and other construction purposes. Phase III included remediation of a large mining encampment, the Industrial Area, adjacent to the Bagega reservoir. More than 1,000 migrant miners had used several dozen crushing/grinding operations that sluiced ore in the regional water supply reservoir. Several thousand tons of highly contaminated tailings were removed from the Industrial Area. Much of this waste material, which regularly exceeded 10% lead, was relocated to area *dabas* (mining sites located remote from the villages) for reprocessing to recover additional gold. The reservoir, which was also highly contaminated, was drained and dredged.

### Disposal

Contaminated soil and mine wastes not designated for reprocessing were disposed of in a series of constructed landfills outside each village. Excavated soil from the landfills was used for clean cover material, fill for ponds, and repair of village roads. Landfills were sited in consultation with ZMOE and village elders familiar with seasonal groundwater levels, water holding capacity, and the structural characteristics of local soils. Landfills were typically 10 m wide and 5 m deep, and extended 30 to > 50 m in length. Those landfills containing contaminated soil < 1% lead were bottom-lined with compacted clay and permanently closed with a 1-m compacted clay cap. Phase III landfills accepting highly contaminated wastes from the Industrial Area were additionally bottom-lined with a polyvinyl chloride (PVC) liner of undetermined thickness purchased and provided by the FMOE. The landfills were delineated by global positioning system (GPS) and closed with permanent monuments dedicated to the children who died in the epidemic.

### Health and Safety

Health and safety protocols for all personnel, workers, and the public were implemented in Phase I and progressively enhanced throughout the cleanup. Training conducted by staff from TG or TerraGraphics International Foundation (TIFO; TG’s nonprofit humanitarian successor) in each village included reviewing remediation and health and safety protocols emphasizing hygiene, proper use of personal protective equipment, construction safety, and decontamination practices (von Lindern et al. 2011). Village health and safety managers ensured best practices were implemented. In Phases II and III, facilities were constructed for workers to shower, change clothes, and clean footwear before leaving the cleanup sites. All laborers participating in excavation or disposal of contaminated materials were provided clean work clothes and dust control masks (model 9211N95; 3M). Lunch was prepared and served to all project participants in specially constructed areas with water for drinking and personal hygiene. All workers were required to wash before lunch. Special hygiene and sample collection precautions were implemented in collaboration with MSF during a concurrent cholera epidemic in 2011. International worker BLLs and malaria monitoring was provided by MSF. Nigerian government worker BLLs were monitored by the ZMOH. Monitoring of village worker BLLs was not conducted. In addition to protecting the workers and communities during remediation, health and safety protocols provided model practices for miners and their families, because many of the cleanup laborers were also engaged in mining.

### Implementation and Advocacy Campaign

Phase I remediation commenced as an emergency response in Dareta and Yargalma, in June 2010. TG and ZMOE developed and implemented emergency remediation plans while MSF and ZMOH established village clinics. The cleanup was conducted by ZMOE, with TG providing technical guidance. Funding and equipment came from Zamfara State, TG, MSF, and Blacksmith Institute (BI; http://www.blacksmithinstitute.org). Security and logistical support for the international contingent was provided by the Zamfara State government and MSF (von Lindern et al. 2011). Phase I was completed in mid-July. Phase II remediation commenced in October 2010 with funding from the United Nations Central Emergency Response Fund, United Nations Children’s Fund, Zamfara State, TG, and BI. Phase II, also conducted by ZMOE with TG oversight, addressed five villages (Abare, Duza, Sunke, Tungar Daji, and Tungar Guru). Remediation activities were completed in March 2011. Preliminary characterization in Bagega was suspended from March 2011 until February 2013 due to lack of funding and security concerns related to the Nigerian presidential election.

MSF initiated blood lead screening and chelation therapy as the remediation program progressed (Greig et al. 2014: Thurtle et al. 2014). However, in May 2011, MSF noted increasing BLLs in a small number of individual chelation patients and suspected lead poisoning in the deaths of two children in remediated villages. Follow-up environmental investigations by TG and ZMOE revealed resumption of mineral processing and recontamination in isolated locations in two villages. Out of concern for recontamination issues, MSF and TIFO initiated an advocacy campaign to persuade the Nigerian federal, state, and local governments to undertake Phase III remediation of Bagega and establish programs to sustain the remedy and promote safer mining. A three-part proposal advocating *a*) MSF establish an outpatient chelation clinic for the children of Bagega, predicated on *b*) remediation of Bagega by the Nigerian federal government under TIFO guidance and certification, and *c*) development of a safer mining program in Zamfara. The proposal was presented to representatives of both Zamfara State and Federal Ministries of Health, Environment, Mining and Solid Minerals, and LGA officials at a conference sponsored by MSF and TIFO, originally scheduled for January 2012 in Abuja, but delayed until May 2012 due to civil unrest in the country (MSF 2012). Numerous delays in the release of cleanup funds postponed the start of Phase III remediation. Significant media attention and pressure came from international and Nigerian nongovernmental organizations (NGOs) (Follow the Money 2012; Human Rights Watch 2012; Murdock 2012). MSF and TIFO maintained permanent staff in Nigeria to conduct negotiations regarding project roles and responsibilities, and to secure the release of funds. In late January 2013, Nigerian President Goodluck Jonathan released $3.2 million to FMOE to undertake the Bagega cleanup, with the bulk of the work to be accomplished by the newly established Zamfara Environmental Sanitation Agency (ZESA), LGA, and local village labor. TIFO provided technical guidance and assistance. Upon certification of completion by TIFO, MSF opened clinics and began BLL testing and treatment. In addition, the Nigerian government allocated $1.1 million to initiate a safer mining program (Abatu 2014).

### Human Subjects, Data Sources and Analyses

Environmental and demographic data and analyses were developed from characterization and construction reports obtained during investigation and remediation activities. Permission to test homes for heavy metal contamination was obtained from residents by Nigerian government representatives by informed consent (TerraGraphics, unpublished data). All environmental and health data presented are de-identified. Numbers of children tested and BLLs were provided by MSF. MSF blood lead data were collected during life-saving medical intervention, and met the standards set by the independent MSF Ethics Review Board for retrospective analyses of routinely collected programmatic data. Review of anonymous, routinely collected programmatic data does not constitute research under the Nigerian National Health Research Ethics Committee guidelines (MSF 2013). CDC blood lead data were collected during medical intervention in collaboration with the FMOH in accordance with the Declaration of Helsinki (Dooyema et al. 2012).

Environmental exposure estimates were developed consistent with the BHSS cleanup model. Every compound in each village was tested by XRF, and residential soil exposure is defined as the average of all surface soil lead concentrations obtained in a compound. The aggregate community soil lead exposure is calculated by averaging mean soil concentrations from all compounds in a village. Percentage reductions and analysis of variance in residential soil exposure were calculated by comparing the pre- and postremediation community mean concentrations (SAS Institute Inc. 2008). Because remediation and soil exposure reductions were prerequisite to chelation treatment, temporal mean first-draw, pretreatment BLL results for children ≤ 5 years of age are presented for context. Temporal means reflect project phases, which were determined by medical urgency, climatic conditions, funding, and logistic factors. MSF BLL screening targeted all children ≤ 5 years in all villages regardless of exposure (Greig et al. 2014; Thurtle et al. 2014).

## Results

**Figure 1:**
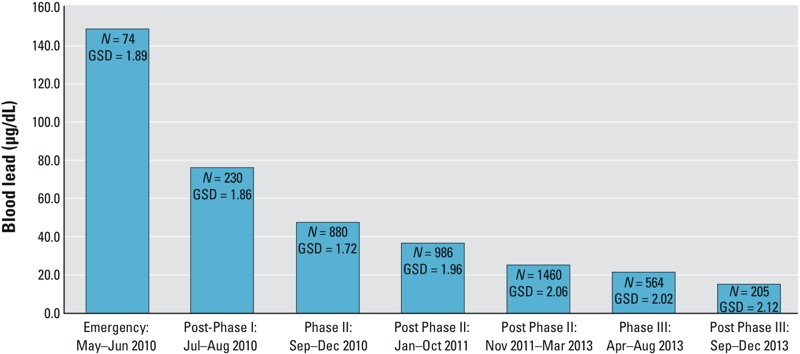
Geometric mean blood lead levels (BLLs) (μg/dL) for first draw (before chelation) for children ≤ 5 years old by cleanup phase (Greig J, personal communication, Operational Epidemiologist, Médecins Sans Frontières). GSD, geometric standard deviation.

Overall, project remediation achieved 77–98% reductions in mean residential soil lead concentrations by village to maximum levels < 400 mg/kg U.S. criteria (U.S. EPA 2008). Soil lead exposures were reduced for an estimated 17,000 residents in eight villages. Testing of all 944 compounds in the eight villages showed pre- and postremediation lead concentrations for individual compounds ranged from 19 mg/kg to 35,380 mg/kg, and 13 mg/kg to 400 mg/kg, respectively. Mean soil concentrations by village ranged from 300 mg/kg to 4,143 mg/kg preremediation and 70 mg/kg to 179 mg/kg, postremediation, with overall means of 1,311 mg/kg and 94 mg/kg, respectively. A total of 820 compounds, 181 common areas, 31 ponds, and the reservoir and Industrial Area at Bagega were remediated (Table 1). Figure 1 shows geometric mean first-draw BLLs for all 4,399 children ≤ 5 years old MSF screened against the chelation threshold (≥ 45 μg/dL) (Greig et al. 2014; Thurtle et al. 2014). Of these 2,349 were provided chelation (Table 2) and MSF observed geometric mean preremediation BLLs decrease from 149 μg/dL to 15 μg/dL over the 4-year cleanup (Figure 1). Phases I, II, and III accomplished 18%, 39%, and 43%, respectively, of the total number of residential compounds remediated, costing an estimated $400,000, $1.9 million, and $3.2 million U.S. dollars, respectively, according to budget estimates presented to the Nigerian government (MSF 2012; von Lindern et al. 2011; TerraGraphics, unpublished data). A total of 27,390 m^3^ of soil and wastes were excavated and disposed of in 14 landfills. Collectively, an estimated 187,000 kg of lead were removed from eight villages and the processing areas (Table 3). More than 43,000 m^3^ of clean soil was imported as replacement fill and cover.

**Table 1:** Pre- and postremediation soil Pb concentrations and range of residential compounds, common areas, and ponds.

Village	Remediation phase	No. of cpds tested	No. of cpds remediated	No. of CAs remediated	No. of ponds remediated	Preremediation mean Pb conc (range) of cpds tested (mg/kg)	Postremediation mean Pb conc (range) of cpds tested (mg/kg)	% of conc reduction
Dareta	I	94	85	13	4	3,436 (40–35,380)	83 (25–252)	98
Yargalma	I	66	63	11	3	4,143 (83–23,296)	179 (25–400)	96
Phase I total		160	148	24	7	3,728	123	97
Abare	II	96	74	20	0	1,343 (43–18,921)	90 (25–400)	93
Tungar Guru	II	38	31	6	1	874 (85–4,446)	83 (23–321)	91
Sunke	II	93	83	38	10	1,119 (19–9,688)	106 (25–400)	91
Tungar Daji	II	78	75	31	10	780 (59–4,952)	72 (25–235)	91
Duza	II	57	57	8	2	300 (24–1,779)	70 (25–209)	77
Phase II total		362	320	103	23	951	86	91
Bagega	III	423	352	54	1	670 (18–20,748)	90 (13–400)	87
Phase I, II, and III total		944	820	181	31	1,311	94	89
Abbreviations: CAs, common areas; conc, concentrations; cpds, residential compounds; Pb, lead.								

**Table 2:** Number of ≤ 5-year-old children provided oral chelation treatment following remediation phases.

Village	Phase I June–Sept 2010	Phase II Oct–Dec 2010	Phase II 2011	Post Phase II 2012	Phase III 2013	Total treated
Abare	10	208	255	86	84	633
Bagega	5	6	0	1	236	243
Dareta	101	182	86	51	53	372
Duza	0	1	53	2	0	56
Sunke	23	81	161	17	25	284
Tungar Daji	0	5	196	23	4	228
Tungar Guru	22	107	24	7	5	143
Yargalma	181	268	70	30	22	390
Total	342	858	845	217	429	2,349
Data from Médecins Sans Frontières (Greig J, personal communication, Operational Epidemiologist, Médecins Sans Frontières).						

**Table 3:** Excavation/disposal volumes (m^3^), Pb concentrations (mg/kg), and total Pb (kg) by phase.

Area	Phase I		Phase II		Phase III		Total disposed	
	Vol (m^3^)	Pb conc (mg/kg)	Vol (m^3^)	Pb conc (mg/kg)	Vol (m^3^)	Pb conc (mg/kg)	Vol (m^3^)	Pb (kg)
Compounds	2,602	3,863	5,183	1,029	3,343	670	11,128	21,502
Common areas	417	2,649	2,418	2,688	1,747	560	4,582	10,471
Ponds/reservoirs	600	11,280	1,380	13,100	700	8,000	2,680	37,144
Process waste	300	32,000	N/A	N/A	8,700	10,000	9,000	117,852
Total	3,919		8,981		14,490		27,390	186,969
Abbreviations: conc, concentration; Pb, lead; vol, volume. Calculation uses soil bulk density of 1,600 kg/m^3^ with 30% bulking factor (http://www.engineeringtoolbox.com/soil-rock-bulking-factor-d_1557.html).								

Phase I remediation included 85 residential compounds and 13 common areas in Dareta, and 63 compounds and 11 common areas in Yargalma. Mean soil lead concentrations were reduced by 98% and 96%, to 83 mg/kg and 179 mg/kg, respectively (Table 1). A total of 3,919 m^3^ of contaminated soils and waste, including 300 m^3^ of 3.2% lead process waste, were excavated and disposed in two landfills (Table 3). MSF initiated blood lead testing concurrent with Phase I remediation. The 74 children screened before and during remediation in May–June 2010 had a mean BLL of 149 μg/dL; 230 children screened in July–August 2010 after Phase I remediation had a mean BLL of 76 μg/dL (Figure 1). MSF provided inpatient chelation to 282 children ≤ 5 years of age from Dareta and Yargalma during Phase I, who were able to return to remediated homes between June and September 2010 (Table 2).

Phase II remediation addressed 320 residential compounds, 103 common areas, and 23 processing ponds in five villages reducing soil lead exposures for an estimated 6,385 residents (von Lindern et al. 2011). Additionally, removal of highly contaminated materials from seven ponds in Yargalma and Dareta, and closure of landfills left open from Phase I were accomplished during Phase II (Table 1). Mean preremediation residential soil lead concentrations in Phase II villages ranged from 300 mg/kg in Duza to 1,343 mg/kg in Abare, and were reduced by 77% and 93%, respectively, postremediation. Five landfills, accommodating 8,981 m^3^ of excavated waste and contaminated soil, were constructed and permanently closed (Table 3). MSF introduced outpatient clinics during Phase II, screening 3,326 children as remediation was completed (Figure 1), and provided chelation to 1,920 children (Table 2).

During Phase II, local government and community activities were initiated to develop technical skills and environmental response capacity in Zamfara. Separate male and female advocacy/environmental health promotion teams were established to facilitate remediation and prevent recontamination. Technology transfer, technical training, and certifications were provided to > 200 ZMOE, LGA, and village personnel. Additionally, nearly one-third of the compounds, common areas, and mineral processing locations in Bagega were characterized, and preliminary design and cost estimates were prepared during Phase II. A 2,000 m^3^ landfill was constructed for Phase III remediation. Supplemental investigations by the CDC and Nigerian authorities during Phase II suggested that artisanal gold mining was occurring in an additional 114 villages in three LGAs. Significant soil contamination (> 400 mg/kg Pb) was found in approximately half of 74 of those 114 villages sampled (Bashir et al. 2014; Lo et al. 2012). Another study revealed metals contamination of surface water and open wells where ore processing had occurred (UNEP/OCHA 2010).

In Bagega during Phase III, 352 compounds, 54 common areas and one pond were remediated (Table 1). A total of 5,090 m^3^ of contaminated soils from compounds and village common areas was disposed of in three landfills. Excavation of the Industrial Area and reservoir produced 8,700 m^3^ and 700 m^3^ of contaminated waste, respectively. These wastes ranged from 2.5% to > 10% lead *in situ* (data not shown). However, the disposed materials averaged 1.0% and 0.8% lead, respectively, because higher concentration materials were removed for reprocessing and the remaining materials were co-disposed with underlying contaminated soils (Table 3). Five kilometers of village roads were graded and capped with laterite soil generated from borrow areas. Approximately 10,000 m^3^ of clean soil used to cap excavated compounds, common areas, the Industrial Area, and newly graded village roads; for construction and brickmaking; and to backfill a large pond that was a drowning hazard for children.

In Phase III, mean soil lead concentrations for all compounds were reduced by 87% from 670 mg/kg to 90 mg/kg. Bagega, with an estimated 7,323 people during 2011, was remediated sequentially in four geographic subareas or quadrants, allowing MSF to commence blood lead screening and outpatient chelation for children in the first quadrant in March 2013. The last quadrant was remediated in July. The mean BLL of 564 children screened in April–August was 25 μg/dL, and for 205 children screened in September–December was 15 μg/dL (Figure 1). Chelation was provided to 236 children with BLLs ≥ 45 μg/dL (Table 2) (Greig et al. 2014). Phase III remediation was accomplished largely by Nigerian personnel trained during Phases I and II. A total of 78 environmental professionals from FMOE, ZMOE, and LGAs directed and supervised all remediation activities including procurement and logistics. More than 300 local community members were trained. Local businesses provided supplies and equipment. Two on-site expatriates participated in Phase III activities, compared with 16 and 28 during Phases I and II, respectively.

Analyses of variance revealed that preremediation soil exposures differed significantly by phase and village (*p* < 0.0001). Mean preremediation soil lead concentrations in Phase II villages (951 mg/kg) tested from October 2010 to March 2011 were 74% lower than May–June 2010 Phase I village levels (3,728 mg/kg) (Table 1). Mean preremediation soil lead levels tested in about one-third of Bagega compounds during Phase II in February 2011 showed concentrations (1,059 mg/kg not shown) (TerraGraphics, unpublished data) similar to those in Phase II villages, but were 670 mg/kg, or 37% lower, by 2013.

## Discussion

Zamfara is a severe example of an evolving trend of the world’s poorest, most remote and vulnerable populations becoming unwitting hosts to environmental and occupational disease. BLLs and mortality rates were unprecedented, exacerbated by risk cofactors that complicated health and environmental response actions (Moszynski 2010; MSF 2010c). Malnutrition and childhood pestilent diseases, including measles, mumps, meningitis, polio, malaria, and cholera, are endemic among these populations (MSF 2010a, 2011; Ogwumike et al. 2012). Health care services are largely nonexistent in the villages. During Phase II, characterization and remediation were conducted in the midst of a cholera epidemic, with > 200 children simultaneously treated for cholera and lead poisoning (Shaffi M, Medical Coordinator, MSF, personal communication, 1 October 2011). Intervention and remediation efforts were hampered by poor infrastructure, as the villages lack electricity and running water, are 1–5 hr travel from the nearest paved roads, and are largely inaccessible in the rainy season. Supplies and equipment had to be procured and serviced from hundreds of kilometers away. Religious and cultural practices required strict separation of adults along sex lines, parallel male and female response teams, and in some situations, suspension of Sharia law. The cash-only economy made the transport and distribution of the large sums of money necessary for payroll, local supplies, and services a logistically complicated and dangerous undertaking. Corruption in the civil government is endemic (Transparency International 2014). Crime and terrorism are constant concerns (U.S. Department of State 2015).

Despite these challenges, cleanup objectives were achieved through adaptation of established U.S. protocols. The soil lead concentrations in Table 1 include every compound in all eight villages and are directly comparable with the 400 mg/kg U.S. standard and are equivalent to the residential soil exposures defined in the BHSS cleanup model. The BLLs in Figure 1 are from nearly the entire population of children ≤ 5 years old. CDC surveyed the Phase I population that MSF screened in May 2010, testing 59% of 345 total surviving children ≤ 5 years old identified in the villages. CDC reported mean BLLs of 130 μg/dL for 86 children in the same two villages (Dooyema et al. 2012), similar to the 149 μg/dL observed by MSF. Population surveys conducted by MSF and TG estimated that 88% of all children ≤ 5 years age in both Phase I and II villages had been screened by January 2011 (von Lindern 2011). As a result, residential soil lead concentrations are an effective exposure metric for these populations, and mean BLLs in Figure 1 are an indicator of the severity of the epidemic at the time.

The emergency measures taken to reduce blood lead levels and environmental exposures (i.e., relocation of mineral processing, remediation, clean drinking water and food sources, chelation) markedly decreased mortality, from 25% in the 6 months preceding May 2010 to < 2.5% by September 2010, significantly reducing the risk of adverse health effects to the resident populations (Dooyema et al. 2012; Greig et al. 2014; Thurtle et al. 2014). Since remediation, new village residents and children born to mothers with low body burden are not experiencing high residential soil lead exposures (i.e., > 400 mg/kg). Similar reductions in other toxic metal concentrations (arsenic, cadmium, and mercury) were achieved simultaneously (Bartrem et al. 2014).

The analysis of variance results suggest preremediation soil lead exposures decreased over time, likely due to both environmental and anthropogenic factors. Phase I and II soil sampling was conducted 1–2 months and 6–12 months following cessation of mineral processing, respectively, with an intervening rainy season; and in Bagega, 9 months and 3 years later. Limited blood lead testing, mortality reports and soil tests conducted in short-term visits to the Phase II villages during Phase I (before the rainy season) suggest that soil lead concentrations and exposures were similar to Dareta and Yargalma in three of the five Phase II villages and in Bagega (TG, unpublished data). In August/September 2010, CDC Nigeria tested 185 children ≤ 5 years old in Bagega; 59% had BLLs > 65 μg/dL (CDC Nigeria, unpublished data). Initially, soil lead concentrations following the relocation of mineral processing varied by village, likely reflecting the intensity of high-lead ore utilization. Environmental factors may have included natural dilution from soil accumulation, runoff during the highly erosive rainy seasons, and wind erosion from seasonal *harmattans* (dust storms). Socially, inadvertent remediation occurred through maintenance, repair, and construction of the mud-brick homes and regular sweeping of floors. Additionally, some families in Bagega remediated their own compounds by scraping the top layer of soil in areas identified as contaminated during the 2010/2011 Phase II characterization effort. These families placed the contaminated soil in sacks and disposed of it at the new ore processing site outside of the village.

These factors suggest that residential soil exposures and BLLs in these villages were much higher in May 2010 than was observed when the remediation and blood lead screening program reached the Phase II villages 6 months later and in Bagega nearly 3 years later. Unfortunately, during the epidemic, nearly every village resident tested showed dangerously high blood lead levels, resulting in deaths, significant adverse health effects among survivors, and continuing body burdens of lead that may require years to equilibrate (Greig et al. 2014; Thurtle et al. 2014). Because the fetal skeleton develops from the mother’s bone store, and newborn BLLs approximate maternal burden, the current population of mothers and young women present an especial risk to future generations (Ettinger et al. 2007; Miranda et al. 2010; Riess and Halm 2007). Potential health effects associated with blood lead levels observed in this epidemic include adverse reproductive and child development outcomes, and irreversible neuropsychological effects ranging from severe brain damage resulting in permanent dysfunction to depressed mental capacity, impairment of nerve function, behavioral and learning problems, loss of quality of life, and inability to participate in or meet village social obligations (Thurtle et al. 2014; U.S. EPA 2006, 2013). There is also a range of possible damage to other organ systems (ATSDR 2007; Caravanos et al. 2013; Lanphear et al. 2005; U.S. EPA 2006, 2013). Because treatment was unavailable for children > 5 years or for adults, entire generations of village residents are potentially suffering lifelong debilitating effects (Needleman et al. 1990; Reyes 2007).

To manage and sustain the remedy and to undertake future cleanup activities, technical capacity was transferred to local entities. Throughout the cleanup, local communities became increasingly aware of the dangers of artisanal mining and measures necessary to protect their families. Several hundred community members were employed and acquired experience in implementing remedial protocols. The Nigerian federal government assumed responsibility to fund remediation and regulate artisanal mining throughout the country. ZESA was created to undertake cleanup activities and regulate pollution from artisanal mining (Sada I, Director General, ZESA, personal communication, 1 February 2011). Local government and emirate officials established committees to address artisanal mining, discourage resumption of dangerous activities, and prevent recontamination in the villages. Nevertheless, the economic contribution of small-scale mining to village livelihood poses a continuing recontamination threat. Alleviating this requires all stakeholders to engage in sustainable region-wide safer mining practices. Institutional controls implemented in 2011–2013 included no longer employing women in ore processing, relocating *dabas* sufficient distances to prevent visits by children, and requiring self-remediation of recontaminated compounds (von Lindern et al. 2011). Wet milling was introduced in 2013–2014 with recommendations to wash and change clothes before going home. Longer-term efforts underway with Nigerian authorities include completing remediation in other villages; developing monitoring, maintenance, periodic reassessment, advocacy, and public health information programs; and establishing safer mining practices (Anka SM, Director, Pollution Control ZESA, personal communication, 3 June 2013; ELI 2014).

## Conclusions

Despite complex challenges, substantial remediation and subsequent medical interventions were accomplished by adapting established health and environmental response protocols to local conditions and capabilities. These efforts, combined with cessation of processing in the villages, provision of clean drinking water, decreased contamination of the food supply, natural attenuation of soil lead concentrations, and behavioral changes, effected 77–98% decreases in soil lead exposures (Table 1) and > 100 μg/dL reductions in mean BLLs (Figure 1). The capacity, authority, funding, and responsibility for the cleanup were transferred to the Nigerian federal, state, and local governments. A cadre of Zamfara State and local government staff was trained to manage and supervise the remediation and undertake sustainable programs to prevent future epidemics. Engagement of villagers and community leaders developed awareness of the dangers of artisanal mining and protective measures families can employ. Sustaining the remedy will require the Nigerians refraining from mineral processing in the villages, and developing and enforcing safer mining practices. This tragic incident and subsequent response demonstrate that, with sufficient political will and modest investment, even the world’s most challenging environmental health crises can be addressed and resolved within the capabilities of the host countries.
